# Phylogenetics and evolution of *Digitaria* grasses, including cereal crops fonio, raishan and Polish millet

**DOI:** 10.1093/aob/mcaf212

**Published:** 2025-10-06

**Authors:** George P Burton, Paolo Ceci, Lorna MacKinnon, Lizo E Masters, Noro Fenitra Harimbao Randrianarimanana, Philippa Ryan, Colin G N Turnbull, Tiziana Ulian, Maria S Vorontsova

**Affiliations:** Royal Botanic Gardens, Kew, Herbarium and Library, Richmond TW9 3AB, UK; Department of Life Sciences, Imperial College London, London SW7 2AZ, UK; Food and Agriculture Organisation of the United Nations, Sub-Regional Office for West Africa (FAO-SFW), Dakar, Zone 6 Parcelle N° 9, Senegal; Royal Botanic Gardens, Kew, Herbarium and Library, Richmond TW9 3AB, UK; Royal Botanic Gardens, Kew, Herbarium and Library, Richmond TW9 3AB, UK; Department of Genetics and Genome Biology, Institute for Environmental Futures, University of Leicester, Leicester LE1 7RH, UK; Royal Botanic Gardens, Kew, KMCC, 3GJV+683, Antananarivo, Madagascar; Mention Biologie et Ecologie Végétales (MBEV), University of Antananarivo, BP 566 Antananarivo, 101, Madagascar; Royal Botanic Gardens, Kew, Herbarium and Library, Richmond TW9 3AB, UK; Department of Life Sciences, Imperial College London, London SW7 2AZ, UK; Royal Botanic Gardens, Kew, Herbarium and Library, Richmond TW9 3AB, UK; Department of Life Sciences and Systems Biology (DBIOS), University of Torino, 8, 10124, Torino, Italy; Royal Botanic Gardens, Kew, Herbarium and Library, Richmond TW9 3AB, UK

**Keywords:** Fonio, food security, agriculture, evolution, *Digitaria exilis*, phylogenetics, domestication, raishan, *Digitaria sanguinalis*, *Digitaria iburua*

## Abstract

**Background and Aims:**

Millet crops in the grass genus *Digitaria* include white and black fonio (*D. exilis* and *D. iburua*), raishan (*D. compacta*) and Polish millet (*D. sanguinalis*), cultivated across West Africa, India and Europe. Fonio and raishan crops are important in supporting food security and subsistence agricultural systems in rural communities, while *D. sanguinalis* is no longer cultivated. These crops are resilient to challenging climates. We aim to produce an integrated study of these crops: a phylogeny of the *Digitaria* genus including all four food species, to identify key crop wild relatives; time-calibrated biogeographic analysis, to investigate the history and evolution of *Digitaria*; and a morphological study to assess the transition between wild and domesticated species.

**Methods:**

We use the Angiosperms353 target-enrichment sequencing approach to produce maximum likelihood and coalescent model nuclear phylogenies for 46 *Digitaria* species, and Bayesian methods to propose an evolutionary and biogeographic history for the genus. Morphology of wild and cultivated species is investigated for spikelets and growth habits using microscopy and SEM imaging.

**Key Results:**

Four distinct evolutionary lineages are found for the *Digitaria* crops, and we identify new close crop wild relatives *D. fuscescens*, *D. atrofusca*, *D. setigera*, *D. radicosa*, and *D. ciliaris*. South and eastern Africa is proposed as a likely origin of early *Digitaria* divergence, with crop lineages diverging from wild relatives around 2–6 mya. Incomplete domestication traits are observed, including the loss of trichomes, but no clear change in appearance for spikelet or abscission zone morphologies.

**Conclusions:**

The knowledge produced in this study about *Digitaria* crop wild relatives will be useful in improving crop traits through targeted breeding and physiological studies, and we also highlight the need for conservation of seed material through programmes working with local partners for these important climate-tolerant indigenous cereals.

## INTRODUCTION

Cereal crops provide the majority of the world’s food, yet are likely to be disrupted heavily by future and present climate change (FAO 2016; Fatima *et al*., 2020). Many underutilized or ‘orphan’ crops are likely to be more tolerant to this change, and can provide resilience in vulnerable food systems that will otherwise be degraded (Mabhaudhi *et al*., 2019; Ulian *et al*., 2020; Santhoshini *et al*., 2025). A selection of these crops have been presented as neglected and underutilized species (NUS; Ulian *et al*., 2020; Animasaun *et al*., 2023), which have experienced far less research and commercial development than major crops, despite their climate-resilient traits and importance to indigenous identity and rural communities. Many of these are millet grasses, including white (*Digitaria exilis*) and black (*D. iburua*) fonio from West Africa. Under-improved crops (including fonio) and crop wild relatives (CWRs) are known to retain allelic diversity which may have disappeared from more heavily domesticated species that experience genetic bottlenecks, and hold the key to preventing diseases, increasing nutrition and providing climate resilience in vulnerable areas (Warburton *et al*., 2006; Hajjar and Hodgkin, 2007; Dempewolf *et al*., 2017; Kashyap *et al*., 2022). The involvement of wild species in crop breeding programmes has been utilized extensively for rice (Stein *et al*., 2018; Zheng *et al*., 2024), wheat (Charmet, 2011) and maize (Warburton *et al*., 2006; Abdoul-Raouf *et al*., 2017). However, for the improved utilization of orphan millets like fonio to be possible and effective, scientists first require a detailed understanding of the taxonomy, evolutionary history and geographic niche of the study species. Knowledge of how grasses have evolved and adapted to climatic conditions in the past can indicate how and why they might respond to challenging future climates.

### *Digitaria* species uses and systematics

In the genus *Digitaria* (Panicoideae: Poaceae) there are four crop species that are or have been important to agriculture systems and providing food for people: white fonio (*D. exilis*), black fonio (*D. iburua*), raishan (*D. compacta*) and Polish millet (*D. sanguinalis*) (Portères, 1955, 1957, 1976; Singh and Arora, 1972; Cruz *et al*., 2016). The genus includes the cultivated pasture grass *D. eriantha* (Pangola grass), common in South Africa, the Americas and Southeast Asia (Parsons, 1972; Tikam *et al*., 2013). *Digitaria* grasses can also have negative economic impacts on agriculture, as the invasive, spreading habit of some wild species (including wild *D. sanguinalis*, *D. ciliaris* and *D. humbertii*) makes them difficult to manage in farm and horticultural settings (Jones *et al*., 2021; Touafchia *et al*., 2023; Randrianarimanana *et al*., 2024), and *Digitaria* grasses are challenging to identify to species level. Although there have been several genomic and phylogenetic studies of *Digitaria*, described below, there has been no integrated approach that considers phylogenetic, morphological and biogeographical analysis of the group through history, or study that includes all four domesticated crop species and close wild relatives. By producing this analysis, we can build knowledge of the close wild relatives of *Digitaria* crops, to facilitate future researchers in conducting research on this genus.

The genus *Digitaria* was first described by Haller (1768), and now contains around 250 species globally (Henrard, 1950; Touafchia *et al*., 2023; POWO, 2025), occupying both temperate and tropical niches. Studies in *Digitaria* systematics include work by Stapf (1915), Henrard (1950) and Vega *et al*. (2009) in morphological classifications. Key traits that have been used to identify species include the shape and size of the upper glume, trichomes on spikelet veins, branching patterns of the inflorescence (technically a synflorescence (Kellogg, 2015), but referred to in this study as an inflorescence for ease of terminology), number of racemes, and number of spikelets in a group (binate = two spikelets, ternate = three or more). These characters are illustrated in Supplementary Data Fig. S1.

Studies in molecular phylogenetics have utilized the combination of ITS sequences and morphology (Lo Medico *et al*., 2017), plastid sequences (Morrone *et al*., 2012) and a mix of Sanger (ITS, *matK*, *rbcL*, *ndhF*, *atpB* and *trnL-F*) and next-generation sequencing (NGS) target-enrichment sequence capture of 353 genes (using the Angiosperms353 bait kit) within a supertree matrix approach (Touafchia *et al*., 2023), to untangle the complex relationships between *Digitaria* grasses, though not wide-scale phylogeny using purely NGS data. Regional taxonomic treatments of *Digitaria* have been conducted in Pakistan and Central Asia, Southeast Asia and Madagascar by Gilani *et al*. (2003), Boonsuk *et al*. (2016) and MacKinnon et al. (In Press), respectively. Minoji and Sakai (2024), in the assembly of a chromosome-level genome for the Asian species *D. radicosa*, also included several of the major clades within a phylogenomic analysis. Throughout these studies several key patterns and traits have remained consistent: the topology includes several major clades distinguished by ternate versus binate spikelet morphology, combined with the presence of spikelets with complex or absent versus simple trichomes (complex trichome morphologies including capitate, verrucose, clavate or hooked, as categorized for *Digitaria* by Touafchia *et al*., 2023). The genus contains many polyploid species, including both fonio species, which were found to be likely allopolyploids, which makes genetic study of the group particularly difficult (Abrouk *et al*., 2020; Minoji and Sakai, 2024). The *Digitaria* clade has also given rise to other morphologically divergent grass genera, including *Anthephora* and *Chlorocalymma* (Touafchia *et al*., 2023).

### Food crops in *Digitaria*

**Fig. 1.**
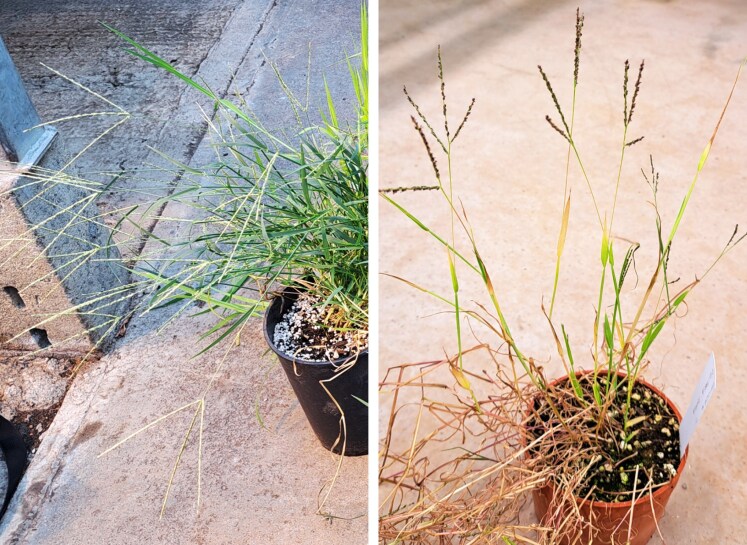
White (left) and black (right) fonio (*Digitaria exilis* and *D. iburua*), cultivated from seed originating in Guinea and Nigeria, respectively, in the Jodrell Glasshouses, Kew. Photo credit and copyright: George P. Burton.

White fonio (*D. exilis*) is commonly grown in West Africa, where it is especially important for both subsistence agriculture in rural areas (Portères, 1976; Cruz *et al*., 2016; Burton *et al*., 2024), and as an increasingly popular export crop (CBI, 2022; Tridge, 2023). It is recognized for its arid climate tolerance, high nutritional value and socio-economic and cultural importance, but also its small grains (1–2 mm in length) and seed husk, which pose post-harvest processing challenges (Portères, 1976; Cruz *et al*., 2016; Animasaun *et al*., 2023). Black fonio (*D. iburua*) is cultivated in small areas of Benin, Togo and Nigeria. It is far less popular than white fonio, and there are few detailed studies available on this rare crop, though Portères (1955) describes the likely domestication of *D. iburua* in the Aïr mountains of Niger before being transported south through to Nigeria, and then to northern Benin and Togo. White and black fonio plants are shown in Fig. 1.

A third *Digitaria* food crop, raishan (*D. compacta*), is a millet cultivated in the Khasi Hills near Shillong (Meghalaya, India), a wet highland environment. Hooker (1897) describes its cultivation in nearby Assam and the Khasi Hills, and Portères reports that in the 1950s it was still under cultivation on ∼100 acres of land in nearby Assam (Portères, 1957). The most detailed published source of information for this crop is a study by Singh and Arora (1972), which includes a theory of how the crop was selected and domesticated from wild *D. cruciata*, including photographs of the crop’s long, unbranching racemes and providing details of its cultivation in tribal communities. This study makes no mention of cultivation in Assam, and since this study there have been no formal published reports of cultivation in Meghalaya. The name *Digitaria cruciata* var. *esculenta* Bor., previously used for the cultivated variety, is now an accepted synonym of *D. compacta* (POWO, 2025), a species with occurrence overlapping wild *D. cruciata* across central and south-eastern Asia. A photograph of raishan grown in the Khasi hills is reproduced from Singh and Arora (1972) in Supplementary Data Fig. S2.

Polish millet (*D. sanguinalis*) is thought to have been cultivated during the 1500–1600 s in Eastern Europe, first intentionally cultivated in a monastery garden in Croatia or Albania, before moving north and expanding into Germany, Hungary and later Poland and Ukraine (Portères, 1955, 1957) before its decline in the mid- to late 1800s. Portères suggests that the first record of this cereal is from 1561, and that it was reported to still be cultivated in Poland in the 1890s; Netolitzky (1914) says that ‘blood millet’ was cultivated in Germany in the 16th century. He mentions no difference between cultivated and wild grains. Henrard (1950) also cites a specimen of the cultivated form of this crop (*D*. *sanguinalis* var. *esculenta*) at the Paris herbarium, which is described to have longer inflorescence racemes and a more robust, erect habit than common *D. sanguinalis*; however, no specimens or detailed records could be found for this study.

### Fonio genetic studies

One of the earliest attempts to investigate the relationship between white and black fonio and their wild relatives using phylogenetic methods was by Hitu *et al*. (1997), who used random amplified polymorphic DNA (RAPD) markers to support that the phylogenetically closest wild relative of white fonio is *D. longiflora* and that of black fonio is *D. ternata*. This relationship was tested and confirmed in later analyses by Adoukonou-Sagbadja *et al*. (2010), Nyam *et al*. (2017) and Ngom *et al*. (2019). Adoukonou-Sagbadja *et al*. (2010) supports a genetic affinity of >92 % between fonio crops and closest wild relatives, and a similar close ancestry between *D. sanguinalis* and the common weed *D. ciliaris*. The production of a chromosome-level annotated genome for *D. exilis* by Abrouk *et al*. (2020) also confirmed the relationship between white fonio and *D. longiflora*, and shared useful insights into synteny shared with other cereal crops.

### Domestication of grass species

Domestication of cereal crops involves phenotypic development or transition based on traits that have been selected by farmers and by cultivation practices (Meyer *et al*., 2012; Fuller *et al*., 2023). Through this ‘accelerated evolution’, wild plants experience selection through many generations of seed collection and re-sowing (Harlan *et al*., 1973). Estimates suggest that transition to early stages of domestication can take as long as 2000 years of active cultivation, but crops display different thresholds of domestication phenotypes over time, dependent on plant pollination and reproductive strategies, and introgression, crossing and cultivation methods (Brush *et al*., 1995; Glémin and Bataillon, 2009; Allaby *et al*., 2017, 2022).

The reduction of seed shattering is a key phenotypic change of cereal domestication: this is a mechanistic change involving the devolution of an abscission zone in the inflorescence, which prevents the grain from easily separating from the rest of the plant before it has reached full maturity (Yu and Kellogg, 2018; Yu *et al*., 2020). This increases the chances of being harvested and cultivated, unlike wild plants which benefit from seed dispersal (Woodhouse and Hufford, 2019). Another phenotype is the reduction of trichomes on the spikelet; in wild plants the presence of trichomes allows the seed or spikelet to be dispersed by the wind and by animals, and helps to establish the seed in the ground. Larger seed size is also a common sign of domestication, reflecting improved seed vigour for successful germination, especially for grasses in arid or difficult environments, and selection for higher yield and seed nutritional content (Fuller *et al*., 2023). By identifying the range of domestication traits shown by a cereal crop like fonio, researchers can strategize routes to trait improvement through breeding and gene editing.

### Aims and scope

Improving our understanding of the evolution of *Digitaria* species will provide valuable knowledge to future cultivation and biodiversity projects. This study aims to identify CRWs within *Digitaria* beyond *D. longiflora* and *D. ternata*, and resolve when, where and how separate crop lineages within *Digitaria* may have evolved.

We construct a well-resolved phylogeny of the genus using target-enrichment sequencing and maximum likelihood (ML) and coalescent-based tree-building methods, with sampling taking into account geography, and clades containing crop domestications. We record and discuss the development of distinct and shared morphologies across the group, including domestication traits. This provides an integrated history of the genus, involving the spread and growth of wild species as an important part of this crop history.

## MATERIALS AND METHODS

### Species sampling and material

Ninety-six samples of 46 species were chosen for DNA extraction and sequencing based on the cladistics and monography presented by Henrard (1950) and Stapf (1915), Lo Medico *et al*. (2017), Vega *et al*. (2009), and Touafchia *et al*. (2023). To approach questions about the evolution of crop species, sample selection was targeted on close relationship and geographic affiliation with key crop species *Digitaria exilis*, *D. iburua*, *D. compacta* and *D. sanguinalis*. *Urochloa ruziziensis* was chosen as the outgroup taxon, within Paniceae. Details of collections involved in the final trees are included in Supplementary Data Table S1.

Samples were primarily collected from dried herbarium specimens available from the K herbarium, with few others from P herbarium (the herbarium codes used are from Index Herbariorum; Thiers, 2025), and others were extracted from silica-dried material from recent fieldwork expeditions. Leaf and inflorescence material was sampled for DNA extraction. Specimens were identified and selected by comparison against Henrard (1950) and against the K herbarium collections, with a focus on type specimens. Type specimens of *D. iburua* and *D. cruciata* var*. esculenta* (to represent *D. compacta*) were sampled at the K herbarium, from native cultivation regions in Nigeria and India, respectively. For material most likely to represent *D. sanguinalis* in its cultivation zone in Europe, a germplasm accession was acquired from the Leibniz Seedbank (IPK) in Germany, which was previously stored and grown annually during the summer in the Alter Botanischer Garten, Gottingen, described as a fast-growing invasive and a descendent of the cultivated variety. This material was grown and sequenced as part of the NERC Environmental Omics Facility (NEOF) *de novo* project NEOF1392 2021, using WGS (included in this study as sample GB999), and will be assembled and published as part of a future ongoing study.

Sixty-four DNA samples and corresponding morphological traits from specimens were recovered and analysed for 46 species: 22 binate, 23 ternate and one with solitary spikelets. Samples were most commonly taken from specimens collected in Nigeria (eight), Madagascar (seven), India (nine) and Ghana (seven). Of all species’ native distributions, 34 occur in Africa, 16 in Asia, 4 in the Americas and 3 in Europe.

### DNA extraction and library preparation

DNA was extracted and isolated from samples using an adapted CTAB method (Doyle and Doyle, 1987), using CTAB, isopropanol and chloroform for purification and magnetic beads for cleaning steps. Target capture sequencing was conducted using the Angiosperms353 target capture probe kit (Johnson *et al*., 2019; Baker *et al*., 2021). Libraries were prepared using indexes from the NEBNext Ultra II DNA Library Prep Kit, and NEBNext Multiplex Oligos from Illumina. Pooled libraries were hybridized with baits from the Angiosperms353 probe kit for 24 h at 65 °C and 12 cycles of PCR. Samples were sent to Macrogen Inc. in Seoul, South Korea, for sequencing using an Illumina HiSeqX platform, for 2 × 150 bp paired-end reads. TapeStation (Agilent Technologies 4200 TapeStation) High Sensitivity D1000 ScreenTapes, gel electrophoresis and Quantus Fluorometer (Promega, USA) measurements were used for validating DNA integrity and quantity throughout the extraction and library preparation process.

### DNA sequence assembly

Raw data reads were trimmed of their adaptors and organized into paired-end and unpaired sequences, and filtered for quality using Trimmomatic 0.39 (Bolger *et al*., 2014). Locus assembly was completed using HybPiper 2.1.2 (Johnson *et al*., 2016) using a DNA target fasta file to capture reads from the Angiosperms353 genes and WGS, using BLASTX v2.5.0 (Camacho *et al*., 2009). The gene coding regions were extracted and distributed into reads per 353 genes, per sample, using HybPiper. Average gene recovery statistics were provided by the hybpiper_stats.py function in HybPiper. Sequences were aligned using MAFFT v7.476 (Katoh and Standley, 2013), then filtered to remove sequences with >30 % missing data within alignments using PhyUtility 2.2.6 (Smith and Dunn, 2008).

### Phylogenetic tree-building

Cleaned gene alignments were (1) used to build a concatenated supermatrix using the -t1 flag ML method, with a GTR + G + I model, in IQ-TREE v2.1.2 (Minh *et al*., 2020), and (2) analysed with IQ-TREE to produce individual ML gene trees based on BIC scores; low support values <0.3 were again collapsed using Newick Utilities v1.6 (Junier and Zdobnov, 2010), taxa with long branches (often due to contamination errors, misalignment or long-branch attraction) removed using TreeShrink v1.3.9 (Mai and Mirarab, 2018), and finally a coalescent species tree was inferred using ASTRAL-III v5.7.7 (Zhang *et al*., 2018). Bootstrap values for ML trees were calculated using 1000 ultrafast replicates in the IQ-TREE concatenated tree, and LPP and quartet scores (QSs) using the -t2 flag in ASTRAL-III for the coalescent species tree.

Visualizations of trees were produced in the R environment v4.4.0 (R Core Team, 2024), using the ggplot2 (Wickham, 2016), ggtree (Yu *et al*., 2017), ape (Paradis and Schliep, 2019), phytools (Revell, 2012) and treeio (Wang *et al*., 2020) packages.

A principal component analysis (PCA) of sequence genetic distance was produced, using AMAS 1.0 (Borowiec, 2016) to concatenate gene alignments from MAFFT, and then a distance matrix of sequences was produced using the dist.alignment function in seqinR (Charif and Lobry, 2007) to calculate a pairwise distance matrix from DNA sequence alignments.

### Time-scaled tree calibration and biogeography

Alignments were input to Sortadate (v2020-12-05; Smith *et al*., 2018), to rank gene clock-likeliness based on tip-to-root variation, bipartition and tree lengths, using the ‘gene-shopping’ method, to avoid computational issues caused by inputting large gene datasets in BEAST analysis. Alignments were concatenated and then split by gene partition using AMAS, to fill missing taxa per alignment. The top 15 ‘best’ genes were input as 15 separate partitions on BEAUti2 (https://www.beast2.org/beauti/).

An uncorrelated lognormal relaxed clock model was used to allow for independent evolutionary rates across taxa. A GTR site model (same as the ML tree) was used, with a *γ* category of 4. Topology (the starting tree) was constrained to the rooted ML tree. A Yule model prior was set, with *U. ruziziensis* set as an outgroup using a mean divergence time of 21 mya (Pessoa-Filho *et al*., 2017; Hackel *et al*., 2018) and a *σ* of 2 (*σ* representing the standard deviation and uncertainty around the mean). The MCMC run was set to 500 000 000 with a burn-in of 50 000 000 (10 %), sampling every 100 000. The.xml control file from BEAUti2 was then run using BEAST2 (Bouckaert, *et al*., 2014). Two independent chains were run and combined. The log files from these BEAST2 runs were analysed using Tracer (Rambaut *et al*., 2018), and tree files with an ESS >200 combined using LogCombiner (https://beast.community/logcombiner). A final maximum clade credibility (MCC) tree with mean node heights was produced using TreeAnnotator (https://beast.community/treeannotator).

The MCC tree from BEAST analysis and a set of binarized species distributions set into five main regions (Americas, West Africa, South and East Africa, Europe and Asia) were used with the program BioGeoBEARS (Matzke, 2013) in R to estimate and illustrate ancestral geographic ranges. Native species distribution data were collected from POWO, GBIF and specimens at K herbarium. The models DEC and DIVALIKE (and +J) were used, and the best iterations were determined using LnL values.

### Ploidy estimation

Ploidy level for samples was estimated using nQuire (v2018-05-05; Weiß *et al*., 2018). Sorted BAM files from the HybPiper gene extractions were input to nQuire, and analysed using the lrdmodel function. Delta log-likelihood values were calculated and used to interpret ploidy (either diploid, triploid, or tetraploid and above). Reported ploidy levels for *Digitaria* species were also accessed from the Chromosome Count Database (http://ccdb.tau.ac.il/), and found in other literature.

### Morphological traits

Morphological traits were extracted from Plants of the World Online (POWO, https://powo.science.kew.org/) and GrassBase (Clayton *et al*., 2016) and verified against specimens and type collections at K herbarium. Spikelet traits were observed using dissection microscopes. Traits collected included growth form (mat-forming, rhizomes or stolons), growth habit (erect, spreading), whether spikelets were binate or ternate, presence and type of trichomes present on spikelets (glabrous, complex or simple trichomes), number of racemes, length of racemes, and length and width of spikelets.

### Imaging

Images of whole plants including inflorescences and roots were taken using an Epson 10000XL scanner at 800 dpi and processed using Adobe Photoshop. Spikelets were imaged using a Leica Z-stacking microscope. *Digitaria* spikelets were drawn to 1.25 mm scale at ×24 magnification by Lucy T. Smith.

SEM was performed using two methods, the first using platinum coating at the Jodrell Laboratory in Kew Gardens and the second using VP-SEM with uncoated spikelets at the Natural History Museum, London. Spikelets were samples from herbarium sheets at K. For SEM the samples were placed on metal stubs and coated with platinum, using a quorum-Q150t ES series sputter-coater. A Hitachi 8230 with a 5 kV electron beam was used to process images. For VP-SEM, uncoated spikelets were placed on metal stubs and observed using a FEI Quanta 650 FEG scanning electron microscope.

## RESULTS

### DNA sequence recovery

DNA recovery statistics from the 353 target genes resulted in capturing an average of 345 genes per sample. Full recovery statistics from HybPiper are provided in Supplementary Data Table S2. Morphological traits, reported ploidy and clade group numbers of key cultivated and wild species are shown in Table 1 and are mapped across phylogenetic trees in Supplementary Data Figs S3 and S4.

**Table 1.** Morphological traits, distribution and reported ploidy of *Digitaria* crop species and CWRs discussed in text

Species	Clade	Distribution	Role	Spikelet	Lower glume	Upper glume	Lower lemma	Ploidy	Reference
*D. exilis*	2	West Africa	Crop	Ternate	Absent	Full spikelet, glabrous	Glabrous	4×	Adoukonou-Sagbadja *et al*., 2007; Abrouk *et al*., 2020
*D. longiflora*	2	Global	Weed	Ternate	Absent	Full spikelet, trichomes	Trichomes	2×, 4×	Hoshino and Davidse, 1988; Adoukonou-Sagbadja *et al*., 2007
*D. iburua*	3	West Africa	Crop	Ternate	Absent	1/2 spikelet, glabrous	Glabrous	4×	Adoukonou-Sagbadja *et al*., 2007
*D. ternata*	3	Global	Weed	Ternate	Absent	1/2–2/3 spikelet, trichomes	Trichomes	4×	Hoshino and Davidse, 1988; Adoukonou-Sagbadja *et al*., 2007
*D. compacta*	5	Asia	Crop	Binate	Small	1/3 spikelet, trichomes	Glabrous	2×, 4×	Mehra, 1982; Löve, 1985; Kumar and Subramaniam, 1987
*D. cruciata*	5	Asia	Weed	Binate	Small	1/3 spikelet, trichomes	Glabrous	2×, 4×	Mehra, 1982; Koul and Gohil, 1991
*D. ciliaris*	6	Global	Weed	Binate	Small	1/2 spikelet, trichomes	Trichomes	4×, 6×	Carnahan and Hill, 1961; Trouin, 1972
*D. sanguinalis*	6	Global	Weed, crop	Binate	Small	1/3 spikelet, trichomes	Trichomes	4×, 6×	Xue *et al*., 1992; Bennett, 1998; Šmarda *et al*., 2014

Distribution is given as native and non-native range, from POWO (https://powo.science.kew.org/).

Ploidy is indicated as 2× (diploid), 4× (tetraploid) and 6× (hexaploid), with references.

### Ploidy estimation

Twenty-eight samples were estimated to be diploid, 22 triploid and 13 tetraploid or above, shown in Supplementary Data Fig. S4. Tetraploid or above samples include *D. iburua*, *D. exilis*, *D. sanguinalis*, *D. monodactyla*, *D. gayana*, and *D. leptorhachis*. Diploid samples include *D. abyssinica*, *D. cruciata*, *D. compacta*, *D. ternata*, and *D. longiflora*. Samples of *D. ciliaris* and *D. sanguinalis* were estimated as both triploid and tetraploid.

### *Digitaria* species topology

**Fig. 2.**
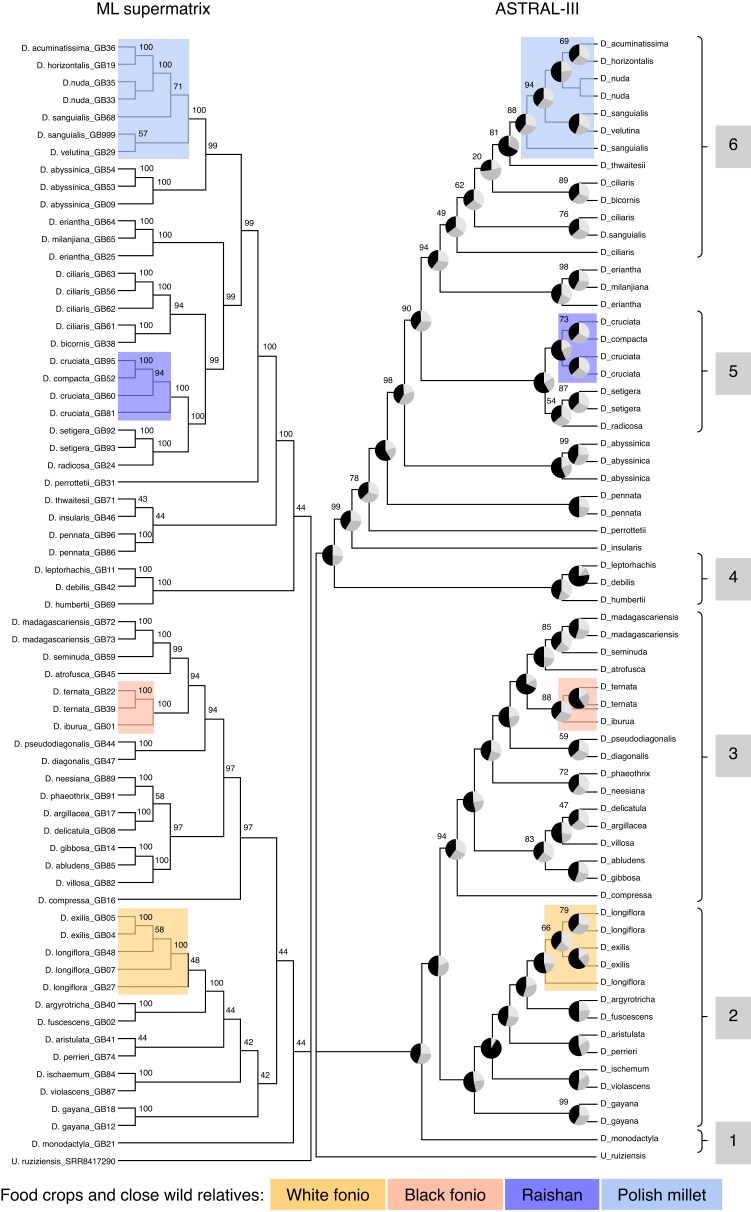
Maximum likelihood supermatrix (left) and ASTRAL-III coalescent (right) phylogenetic trees of *Digitaria* species. Bootstrap scores are displayed on the ML tree, and QSs and pies show primary (black), second (medium grey) and third (light grey) gene tree topology agreement. Species with cultivated food species and close wild relatives are highlighted: fonio species in orange, and raishan and Polish millet species in blue. Clades are numbered 1–6 for discussion: 2–3 are ternate species and 4–6 are binate. Not all species are included within numbered clades due to discrepancy between trees.

**Fig. 3.**
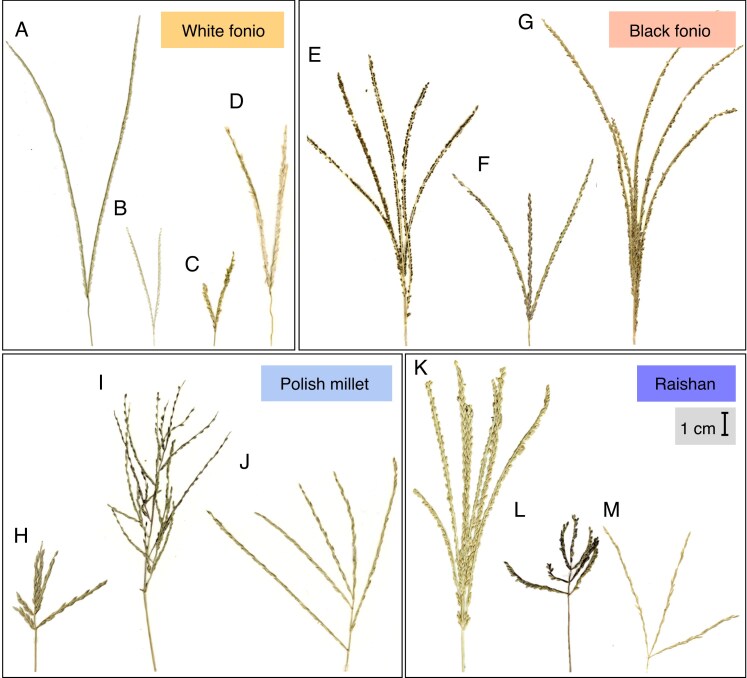
Photo scans of *Digitaria* inflorescences from herbarium specimens, representing the four main cultivated food crops and closely related wild relatives. Species shown are: (A) *D. exilis* (DP 1001), (B) *D. longiflora* (R.A. Farrow 112a), (C) *D. fuscescens* (J.B. Hall 3304), (D) *D. argyrotricha* (Greenway 10819), (E) *D. iburua* (DP 1010a), (F) *D. ternata* (Tuley 1547), (G) *D. atrofusca* (A. Falannigui 25), (H) *D. sanguinalis* (Stewart 26651), (I) *D. velutina* (Hatch 4296), (J) *D. ciliaris* (Gledhill 200), (K) *D. compacta* (Hooker & Thomson s.n.), (L) *D. cruciata* (Stainton, Sykes & Williams 3989) and (M) *D. radicosa* (Vorontsova 2116). Scale bar = 1 cm; all images are to same scale.

There is similar topology for *Digitaria* species presented by the ML and coalescent method trees produced by IQ-TREE and ASTRAL-III, respectively, shown in Fig. 2. In both trees, there is a split into two large clades: clades 1–3 with almost exclusively ternate species (*D. monodactyla* in clade 1 being the only binate species) and clades 4–6 with exclusively binate species. Species inflorescence morphology for each food crop clade is shown in Fig. 3.

#### Clade 1: *Digitaria monodactyla*

*Digitaria monodactyla* is the first-diverging species in the wider ternate group, though it is itself binate. The location of this primary divergence is the same on both trees with high ASTRAL-III support values >99 % and gene tree congruence for first topology.

#### Clades 2 and 3: white and black fonio and relatives

Within clades 2 and 3 the two different fonio crop species emerge into separate well-supported clades in both trees: clade 2 with white fonio (*D. exilis*) and relatives *D. longiflora*, *D. argyrotricha* and *D. fuscescens* (this sister clade supported by 100 % ML bootstrap and QS), and clade 3 with black fonio (*D. iburua*) and relatives *D. ternata*, *D. atrofusca* and *D. madagascariensis* (sister clade supported at 94 % ML and 100 % QS). On the ASTRAL-III tree, low gene tree congruence in clade 2 separates multiple samples of *D. longiflora* and *D. exilis* (58 % ML, 66 % QS). One *D. longiflora* sample from Tanzania (GB27) is observed to be outside the clade containing West African fonio and other *D. longiflora* samples (48 % ML, 100 % QS). Support for the separation between *D. iburua* and *D. ternata* in clade 3 is strong at 100 % ML, and 88 % QS.

#### Clade 4: *Digitaria leptorhachis*, *D. debilis* and *D. humbertii*

A clade with *D. leptorhachis*, *D. debilis* and *D. humbertii* contains the first-diverging species among the binate species (not including *D. monodactyla*). This is congruent in both trees, with 100 % ML and QS support, and especially strong gene tree agreement in the ASTRAL tree for the relationship between *D. debilis* and D*. leptorhachis*.

#### Clade 5: raishan and relatives

Both trees place the Indian crop raishan (*D. compacta*) in a well-resolved clade with relatives *D. cruciata*, *D. setigera* and *D. radicosa*, with identical topology between trees and high ASTRAL-III support for the difference between *D. cruciata* samples and relatives *D. setigera* and *D. radicosa* (100 %) and for the whole clade (90 %). This is similarly high in the ML tree, with 94–100 % support values for the whole clade and within.

#### Clade 6: Polish millet, *D. ciliaris* and relatives

The placement of *D. sanguinalis* samples is poorly supported in the ML tree (57–71 %), but more confidently in ASTRAL-III (84–94 %), where two samples from India (GB68) and Germany (GB999) appear more closely related to *D. nuda* and *D. velutina*, respectively.

The most discordant relationship between trees is the placement of species *D. abyssinica*, *D. eriantha* and *D. ciliaris*. While the ML tree presents clade 5 and *D. ciliaris* as well-supported sister groups (99 %), with samples of *D. ciliaris* from India, Brazil and Africa forming a single clade in the ML tree (94 %), the ASTRAL-III tree splits the *D. ciliaris* samples with low support (20–62 %), placing them between the *D. eriantha* and *D. sanguinalis* clades. *Digitaria eriantha* itself is well supported as a clade with *D. milanjiana* at 99–100 % ML, and 94–100 % QS, and appears to be closely related to *D. ciliaris* in both trees. A clade of *D. abyssinica* contains samples from across Africa and South America, all supported in the same clade together in both trees (99–100 %); however, it appears to be more closely related to *D. sanguinalis* in the ML tree (99 %), while its placement outside of both *D. compacta* and *D. sanguinalis* clades in the ASTRAL-III tree is similarly well supported (98 %).

#### Genetic distance matrix

**Fig. 4.**
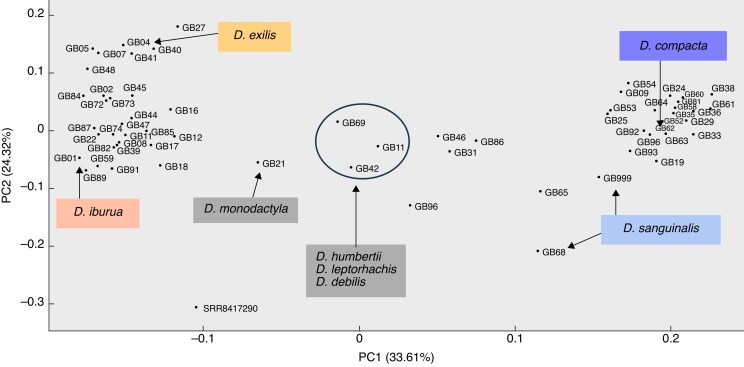
PCA plot of genetic distance between *Digitaria* sample alignments. Colours correspond to crop clades shown in Fig. 2; key species are indicated in the labels, including early-diverging *Digitaria* ternate species *D. monodactyla*, and the binate group is circled. Sample SRR8417290 is the outgroup species *Urochloa ruziziensis*.

A PCA of genetic distance between concatenated sequence alignments is shown in Fig. 4; the species *D. monodactyla*, *D. debilis*, *D. humbertii* and *D. leptorhachis* appear to be the closest to the outgroup *U. ruziziensis*, and the most separate and distant from either major binate or ternate clades. These distinct divergences are well supported in both ML and ASTRAL-III trees, which share the same main topologies in gene congruence.

### Evolution and biogeography

**Fig. 5.**
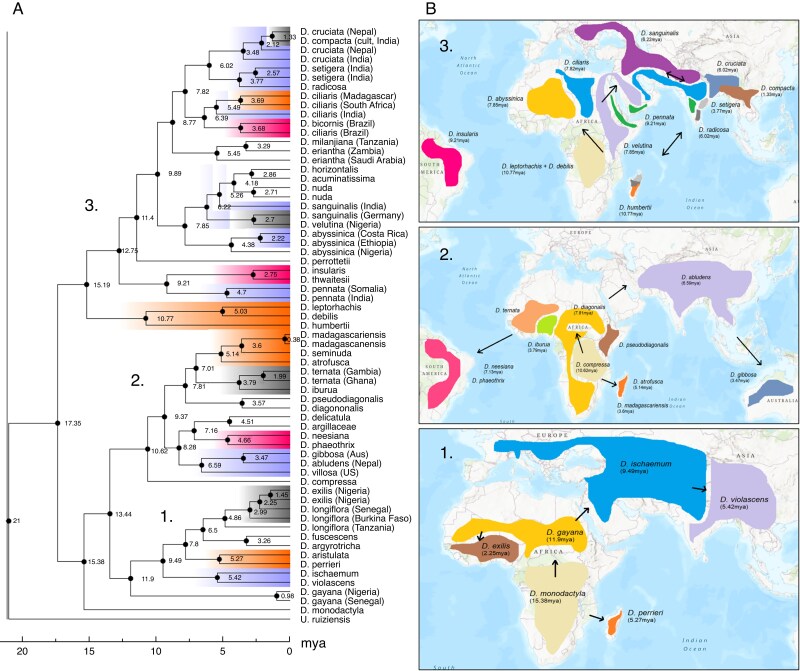
Bayesian (BEAST2) time-calibrated phylogeny of *Digitaria* species and likely dispersal routes. (A) Maximum clade credibility (MCC) tree, where clades are coloured by dispersals to Madagascar (orange) and the Americas (pink). Cultivated food species and relatives are highlighted in grey. (B) Likely routes of dispersal, corresponding to clades numbered in the MCC tree. Maps are based on overlapping native extant species distributions, from POWO (https://powo.science.kew.org/).

The results of the BEAST2 Bayesian analysis are shown in Fig. 5. The earliest-diverging extant *Digitaria* are African natives *D. monodactyla*, *D. compressa* and *D. gayana* from mostly ternate clades 1–3, and *D. debilis*, *D. leptorhachis* and *D. humbertii* in binate clade 4. Both *D. exilis* and *D. iburua* are estimated to have diverged from wild relatives around 2–3 mya. *Digitaria sanguinalis* diverged from *D. velutina* (north Africa) around 2.7 mya, though an earlier divergence into India is dated at 6.22 mya. The specimen of *D. compacta*, cultivated in India, diverged from wild *D. cruciata* more recently at 1.33 mya, and both species from the clade containing *D. radicosa* and *D. setigera* around 6 mya.

Biogeographical analysis by BioGeoBEARS is shown in Supplementary Data Fig. S5, which supports an ancestor of *Digitaria* in south-eastern Africa (DEC model LnL = −167.38, DEC + J LnL = −162.34). This corroborates the BEAST results, where the earliest-diverging *Digitaria* (including *D. monodactyla*, *D. gayana*, *D. compressa*, *D. leptorhachis* and *D. debilis*) all have native distributions that overlap in south and eastern Africa.

### Domestication

**Fig. 6.**
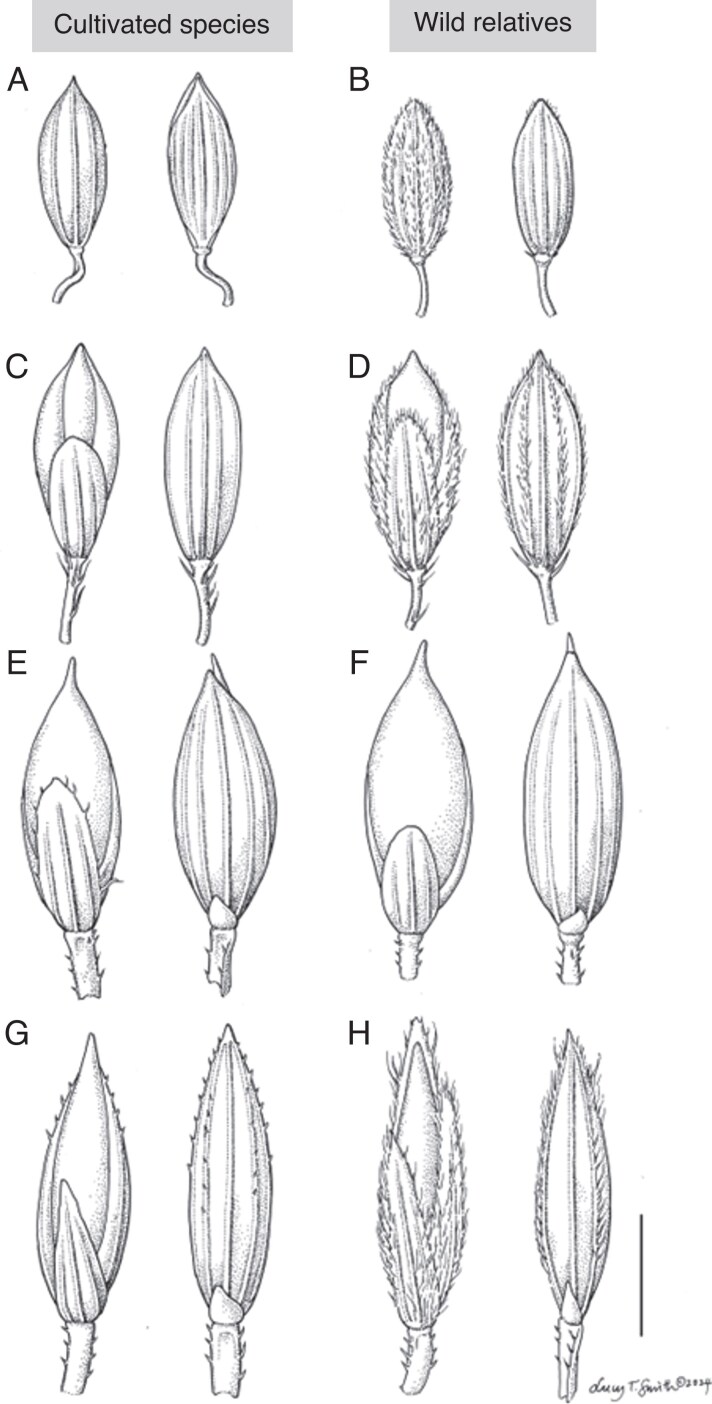
Illustrations of *Digitaria* spikelets, representing the four main cultivated food crops and CWRs. (A) *D. exilis* (Clarke s.n), (B) *D. longiflora*, (C) *D. iburua* (Lamb 54), (D) *D. ternata* (Schimper 76), (E) *D. compacta* (Hooker & Thomson s.n.), (F) *D. cruciata* (Royle s.n), (G) *D. sanguinalis* (Stewart 26651), (H) *D. ciliaris* (Lindeman & Haas 459). Drawn by Lucy Smith at Kew Gardens. Scale bar = 1.25 mm at ×24 magnification.

Illustrations of *Digitaria* crop and wild species spikelets are shown in Fig. 6. There appears to be a loss of trichomes for cultivated fonio species, though it is not fully complete. *Digitaria longiflora* has trichomes present on the spikelet and pedicel that are not present on *D. exilis*. Trichomes are present on the spikelet and pedicel of *D. ternata*, and on *D. iburua* there are no trichomes on the spikelet but they are still present on the pedicel. Trichomes are present on the pedicel of both *D. compacta* and *D. cruciata*, and only slightly discernible on some specimens on the spikelet. On *D. sanguinalis* complex trichomes are very noticeable across the whole spikelet and pedicel. Close relatives *D. ciliaris* and *D. velutina* (not pictured) are similarly pubescent.

Regarding the size of the spikelet, from average measurements provided by GrassBase (Clayton *et al*., 2016), *D. exilis* has an average spikelet length of 1.7–2 mm versus 1.2–2 mm for *D. longiflora*, and *D. iburua* has ∼2 mm versus 1.8–2.7 mm in *D. ternata*. Although there are very few samples of cultivated *D. compacta* available, the average spikelet length is recorded as ∼ 2 mm versus 2–3.5 mm in *D. cruciata* and 2.5–3 mm in *D. radicosa*. *Digitaria sanguinalis* has an average of 2.5–3.5 mm, which is smaller than 1.5–2.1 mm in *D. velutina*, but similar to 2.5–4 mm for *D. ciliaris*. Visually, the crop species do not appear any larger than wild species; thorough future morphometric studies should be conducted on a wide range of material to confirm this.

**Fig. 7.**
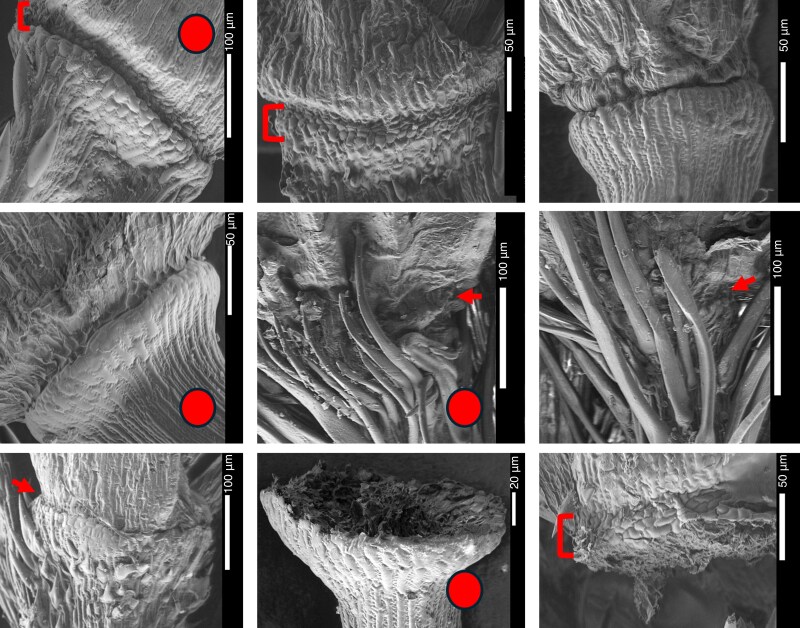
Scanning electron microscope photos of *Digitaria* abscission zones in cultivated and close wild relatives, captured at Kew Gardens and the Natural History Museum (UK). Red circles indicate cultivated species. Arrows and brackets indicate the abscission zone area. Species from top left to bottom right are *D. compacta* (Hooker & Thompson s.n.), *D. cruciata* (Royle s.n), *D. longiflora* (Merklinger 65), *D. exilis* (Burton 15), *D. iburua* (Lamb 54, type), *D. ternata* (Hall 636), *D. sanguinalis* (Santos 1844), *D. exilis* (Thomas 1485) and *D. sanguinalis* (Peterson 23883).

The abscission zones for *Digitaria* species are shown in Fig. 7 (cultivated and CWRs) and Supplementary Data Fig. S6 (wild species). The abscission zone is evident below the whole spikelet, shown by comparison of the complete spikelet and removal, where an empty pedicel is shown. On the abscission zone itself, a layer of specialized cell structures appears to be arranged horizontally in squares, perpendicular to the vertical, long striations in the pedicel likely representing lignification patterns. For small millets, looking at cell arrangements is a good alternative for smooth versus rough appearances in archaeological studies. The same abscission zone cell patterns are seen in both wild *D. cruciata* and cultivated *D. compacta*, as is the ‘clean’ line pattern in both *D. longiflora* and *D. exilis*, and in *D. iburua* and *D. ternata*. The ‘horizontal cell’ pattern is only observed for binate species in *D. sanguinalis*, though this may be due to inconsistency of age between specimens. There appears to be no easily noticeable or consistent phenotypic difference between the wild and cultivated species. In Supplementary Data Fig. S6 an array of wild *Digitaria* species is shown for comparison, presenting the concave and convex sides of the pedicel base and abscission zone layer, which follows the same patterns as seen in cultivated and CWR species.

## DISCUSSION

### *Digitaria* species topology

The structure of the nuclear trees and general clade topologies concur with those of the trees presented by Touafchia *et al*. (2023) and Minoji and Sakai (2024). The topology is also supportive of the systematics proposed by the morphometrics of Vega *et al*. (2009) and the Sanger phylogenies of Lo Medico *et al*. (2017) and Morrone *et al*. (2012). However, these trees are the first presentation of *Digitaria* using a high number of NGS low-copy target nuclear genes, a wide range of species sampling both geographically and across all major clades, and including all four known species domesticated for food cultivation, and their close wild relatives. Touafchia *et al*. (2023), for example, only used 17 *Digitaria* NGS sequences, and did not include white or black fonio or raishan samples in their phylogeny.

#### Clade 2: white fonio and relatives

The closest relative of *D. exilis* is *D. longiflora*, following previous studies including Adoukonou-Sagbadja *et al*. (2010) and Abrouk *et al*. (2020). A sample of *D. longiflora* from Tanzania, the only one sampled from outside of West Africa, is also seen to be less closely related to *D. exilis* in both trees, supporting that *D. exilis* diverged from *D. longiflora* in West Africa, and is more closely related to populations there. The two species are similar in morphology and often grown in close proximity in cultivated fields across West Africa (Burton *et al*., 2024), though *D. exilis* has lost its trichomes, has increased raceme number and length, and has abandoned the rhizomatous growth habit.

The next set of closely related species are *D. fuscescens* and *D. argyrotricha*, the former of which is native to West Africa, and the latter to the coast of East Africa. All four species have maintained a strong v-shaped finger inflorescence shape with no or rare auxiliary branching (Fig. 2). *Digitaria exilis* is the only species that commonly has more than two racemes, though this varies between landraces, and the racemes are usually still connected at a single node. The morphology of *D. fuscescens* is similar to that of *D. longiflora* in the short racemes, and has similar ‘weedy’ invasive traits, such as a spreading habit, stoloniferous roots and small inflorescences. *Digitaria argyrotricha* has longer racemes with larger spikelets, and these are covered with a dense coat of trichomes. *Digitaria gayana* is the earliest-divergent ancestor in this lineage, an African native that varies between a v-shaped and a sometimes more branched inflorescence, and spikelets also with dense trichomes.

White fonio has not become completely indistinguishable from its close relatives; its racemes are longer, but it still experiences lodging and shattering. Its lineage in invasive and drought-tolerant African grasses is likely to offer unexploited potential, though it otherwise suffers from noticeably incomplete domestication.

Samples of the two fonio crops *D. exilis* and *D. iburua* are estimated to be tetraploid (2*n* = 36), corroborating the previous study by Adoukonou-Sagbadja *et al*. (2007). Close relatives *D. longiflora* and *D. ternata* were estimated to be diploid, contrary to previous studies that suggest both are tetraploid (Adoukonou-Sagbadja *et al*., 2007; Abrouk *et al*., 2020). Only one diploid accession of *D. longiflora* (2*n* = 18) has been reported, by Hoshino and Davidse (1988). Close relative *D. fuscescens* is estimated here as tetraploid, and has been reported as both diploid and tetraploid in Gould and Soderstrom (1967, 1974).

#### Clade 3: black fonio and relatives

The close relatedness of *D. iburua* and *D. ternata* has been reported since Hitu *et al*. (1997), and is supported again here. A sister clade containing the common African weed *D. atrofusca* is also presented. All three species share a common morphology: multiple secondary branches from the main rachis and black upper florets, also shared with their earliest-diverging species in the clade, African *D. compressa*. Between wild species and *D. iburua* we observe the development of thick culms and robust racemes, and the loss of spikelet trichomes. *Digitaria ternata* and *D. atrofusca* overlap with *D. iburua* in its restricted cultivation area in Nigeria, and also both occur across West and south-eastern Africa. It is likely from this, their shared morphologies and phylogenetic similarity that there were several speciation events within this cluster of species in West Africa as early as 8 mya.

The documentation of black fonio cultivation and subsequent research on its traits and genetic history is particularly scarce, and recent research aimed at improving knowledge about *D. iburua* often rely on germplasm provided by seed banks in the USA, Togo, Benin and Nigeria. However, due to low germplasm quantities stored at these seed banks, very limited germplasm could be obtained for this and other related studies, from a single accession collected in the 1960s. The authors were fortunately able to collaborate with Dr Abubakar Bello and Ishaq Muawiyya from Umaru Musa Yar'adua University, Nigeria, and early surveys from their work confirm that black fonio is still being cultivated in the Jos Plateau region. The scarcity of available germplasm material for black fonio is concerning, and more collections should be made to enable future research and conservation. Future research with this crop will benefit from including material not only from *D. ternata* but also *D. atrofusca* in their studies. Although *D. iburua* also suffers from apparent incomplete domestication in its small shattering grain, its robust growth habit and multiple racemes make it maybe even more attractive than *D. exilis* for breeding programmes, if it can be conserved, and promoted alongside improved access to dehusking and processing machinery.

#### Clade 5: raishan and relatives

*Digitaria compacta* is confirmed to be closely related to the wild *D. cruciata*, with a sister clade including *D. radicosa* and *D. setigera*, all Asian grasses. Morphology-based cladistics of *Digitaria* from Pakistan and Central Asia in a study by Gilani *et al*. (2003) corroborate these findings, predicting a close relationship between *D. cruciata* and *D. sanguinalis*, followed by *D. ciliaris*, *D. setigera* and *D. radicosa.* There is a stark development from *D. cruciata* to the cultivated *D. compacta*: though they have the same inflorescence structure with opposite secondary branchings from the same rachis node (in a star shape), the cultivated form has long racemes with more grains, and a robust root-to-culm growth form. The inflorescence structure of the sister clade which includes *D. setigera* and *D. radicosa* are also similar in their parallel inflorescence branchings. Although all three species are common from Afghanistan eastwards, *D. radicosa* and *D. setigera* also occur in Madagascar and other areas of south-eastern Africa, across which dispersal events may have taken place.

The recently published chromosome-level analysis of relative *D. radicosa* in Minoji and Sakai (2024) included an analysis of synteny between *D. radicosa* and other crops, finding strongly similar syntenic blocks between *D. radicosa* and *D. exilis*, and with rice (*Oryza sativa*), including a diverse range of NLR (resistance gene analogue RGA5) genes to protect from blast fungi. *Digitaria radicosa* is likely to be a useful CWR species in future research.

Both *D. cruciata* and *D. compacta* are estimated to be diploid in this study, and have been previously reported as diploid and tetraploid (Mehra, 1982; Löve, 1985; Kumar and Subramaniam, 1987; Koul and Gohil, 1991). Close relative *D. radicosa* is estimated to be tetraploid in this study, but was found to be exclusively diploid in a chromosome-level genome assembly and survey by Minoji and Sakai (2024) .

Raishan has been far more neglected in terms of academic study, commercialization and policy initiatives than the African fonio millets. As part of this study, the authors were able to contact and collaborate with researchers at the Botanical Study of India, and with Dr Meera Das and Fullmerries Puwein at the North-Eastern Hill University, in the Shillong region, Meghalaya, India. Communication with rural farmers in the Khasi Hills has confirmed that the crop was still being cultivated in early 2024, though it is rare and under threat, and being replaced by varieties of higher-yielding finger millet (*Eleusine coracana*). Raishan has a host of traits that may be beneficial and interesting for further study, including its large grains and long, numerous racemes, and may provide useful information to breeding programmes to benefit food security in both West Africa and India, if international effort is made to protect this species. Recent Indian government initiatives, including the UN’s International Year of Millets 2023, made numerous references to raishan in advertising material and policy documents, but there has so far been little effort to accelerate research or develop initiatives for its conservation and promotion. Seed-banking led by local indigenous people would be a particularly effective method for its protection and conservation.

#### Clade 6: Polish millet, *D. ciliaris* and relatives

There is noticeable discrepancy in placement of *D. ciliaris* and relatedness to *D. sanguinalis* and *D. compacta*. Henrard (1950) makes observations about the blurred and overlapping suite of characters for these species, and their overlapping occurrences throughout Asia, Europe and northern Africa. Previous study of these species has supported two discrete, separate species as *D. sanguinalis* and *D. ciliaris*, but with closely shared ancestry (Adoukonou-Sagbadja *et al*., 2010).

*Digitaria sanguinalis* is estimated as both triploid and tetraploid in this study, corroborating other literature (Šmarda *et al*., 2014), and also reported as hexaploid and above elsewhere (2*n* = 54; Xue *et al*., 1992; Bennett *et al.*, 1998). *Digitaria ciliaris* is similarly estimated to be triploid and tetraploid here, and as tetraploid and hexaploid in the literature (Carnahan and Hill, 1961; Trouin, 1972), and so both are polyploid species.

The closely related species *D. sanguinalis*, *D. ciliaris*, *D. nuda* and *D. horizontalis* are known to frequently occupy fields together (da Silva *et al*., 2024), and so there may be ongoing or historical hybridization. It may be useful to consider clade 6 containing these species in the ASTRAL-III tree as a broad taxonomic complex. Somewhere in this complex, a landrace precursor in populations selected as Polish millet would have been foraged and cultivated in Europe, likely a hybrid between several of the species in this clade. This contrasts with the well-resolved and distinct lineages of the other *Digitaria* crops. As *D. sanguinalis* is no longer cultivated as a food crop in Europe, it is difficult to untangle this complex.

### Polyploidy

Inconsistencies between chromosome count studies in the literature and some of the ploidy estimations in this study (including around *D. exilis* wild relatives and *D. radicosa*) show how challenging it can be to conduct phylogenetic studies of polyploid species using NGS target-capture sequencing data (Griffin *et al*., 2011; Dufresne *et al*., 2014; Kyriakidou *et al*., 2018). The accuracy of estimations made by nQuire analysis has also been questioned (T. Sakai, Japan, Kyoto University, pers. comm.). There is certainly scope for future research to reassess the complex interactions of *Digitaria* species topologies, crop domestication and polyploidy in panicoid grasses, using recently developed methods such as that of Masters *et al*. (2024) for *Brachiaria* grasses using allele phasing, and phylogenetic network analysis using tools including PhyloNetworks to detect possible reticulation and hybridization events (Solís-Lemus and Ané, 2016; Solís-Lemus *et al*., 2017).

### Evolution and biogeography

From species distributions and BEAST node age estimation it is predicted that early groups of *Digitaria* diverged at least 15–17 mya in central or south-eastern Africa, during the Miocene period. This corroborates with estimated divergences of Anthephorinae (the subtribe within which *Digitaria* belongs) dated to a mean age of 18.43 mya by Gallaher *et al*. (2022), with a likelihood of ∼21 % having originated in the Afrotropics region. The origin of the tribe Paniceae is estimated to 29.96 mya with a 38 % likelihood of origin in the Afrotropics, in the same study. Hackel *et al*. (2018) estimates the divergence of Paniceae at around 22 mya, and the split between binate and ternate *Digitaria* at around 15 mya (similar to 15–17 mya in this study). Africa has generally been suggested as an area of early divergences for many grass lineages (Gallaher *et al*., 2022; Peterson and Soreng, 2022), especially C4 grasses, as well as for Poaceae as a whole (Bouchenak-Khelladi *et al*., 2010), which corroborates our results here.

In the phylogenetic trees, the PCA the and biogeographic and time analyses, it appears that *D. monodactyla*, *D. debilis*, *D. leptorhachis*, *D. humbertii* and *D. compressa* are the earliest-diverging and most genetically dissimilar to other later-diverging *Digitaria*, and are all African native species. *Digitaria monodactyla* is one of the only binate species within the ternate group, and has uncommonly single, spicate racemes. *Digitaria monodactyla* and *D. compressa* also have fire-adaptive culm crowns, adapted to fire-regime lifestyles in seasonal savannah open grasslands in south-eastern Africa (see photographs of growth forms in Supplementary Data Fig. S7). The mid- to late Miocene saw aridification of parts of south-eastern Africa that shaped the evolution of plant species (Osborne, 2008; Pokorny *et al*., 2015), and in the later period allowed the expansion of savannah and grasslands (Sepulchre *et al*., 2006). The formation of these grasslands saw the dominance of C4 grassland species in these drier climates between 20 and 10 mya (Edwards *et al*., 2010; Peppe *et al*., 2023), which is thought to have coincided with ‘enhanced fire activity’ (Hoetzel *et al*., 2013; Karp *et al*., 2018). This expansion of C4 grasses, an increasingly arid climate and fire activity would have both shaped and supported the diversification of *Digitaria* grasses, including ancestors of the early-diverging extant species *D. monodactyla* and *D. compressa*, and the spread of both the ternate fonio lineage to the west and the binate raishan and Polish millet lineages north to Europe and Asia.

In other phylogenetic studies including *Digitaria*, several species included in other genera are embedded exclusively within the *Digitaria* binate clade, and not among the ternate species clades (Hackel *et al*., 2018; Touafchia *et al*., 2023; Grass Phylogeny Working Group III, 2025), including *Chaetopoa taylorii*, *Chlorocalymma cryptacanthum*, *Taeniorhachis repens*, *Anthephora pubescens*, *A. cristata*, *Tarigidia aequiglumis*, *Stereochlaena caespitosa* and *Baptorhachis foliacea*. These species are native to dryland African grasslands (Bouchenak-Khelladi *et al*., 2010; Grass Phylogeny Working Group III, 2025; POWO, 2025). *Chaetopoa taylorii*, *C. cryptacanthum*, *B foliacea* and *A. pubescens* also all have single racemes (similar to the spicate inflorescence of *D. monodactyla*). These species provide an ‘African grassland’ core to the otherwise quite widely distributed binate species, and possibly represent a closer relationship to the earlier-diverging *Digitaria* in clades 1 and 4 from south-eastern Africa, with close morphological similarity and ecological habitat.

From the few native species sequenced as part of this study, *Digitaria* appears to have migrated to Madagascar from as late as 10–15 mya, with the separation of *D. humbertii* (endemic to Madagascar) from *D. debilis* and *D. leptorhachis* (occurring in mainland Africa, and *D. debilis* also occurring in Madagascar). *Digitaria humbertii* is a grazing-adapted grassland species, with rhizomes, spreading and mat-forming; its sister species *D. debilis* and *D. leptorhachis* occupy a similar ecological niche, but are more erect in their growth habit. In a study by Randrianarimanana *et al*. (2024), ethnobotanic interviews with farmers across three regions in central and eastern Madagascar cited *D. humbertii* as the most troublesome grass weed in agricultural settings, followed by *D. longiflora*, which also has a similar growth habit to *D. humbertii*. Other *Digitaria* species, including *D. ternata*, *D. ciliaris*, *D. sanguinalis* and *D. debilis*, were also reported. This ‘weedy’ habit is seen in early- and late-diverging species on mainland Africa and Madagascar, and this phenotype was shown by Touafchia *et al*. (2023) to not be specific to any clade across the genus, and to have instead evolved convergently many times. Hackel *et al*. (2018), in investigating the origin and diversity of C3 and C4 grass evolution in Madagascar, comments that endemic C4 panicoid grasses are likely to have appeared during the Miocene, supporting the formation of the *D. humbertii*–*D. debilis* clade during this period.

### Fonio domestication

Despite a long history of evolution, *Digitaria* crops do not show significant developments that make them overly distinct from their wild relatives: there is no distinct abscission zone change to suggest a strongly evolved non-shattering phenotype, the grain sizes overlap, and there is only complete loss of trichomes in *D. exilis* (the most commonly cultivated species). In a study of 203 global food crops by Meyer *et al*. (2012), the term semi-domesticated’ is used to describe crops that have achieved only partial differentiation from wild species, and seems relevant to apply to *Digitaria* crops.

The only study to consider fonio abscission zones (a key mechanism for regulating shattering) in depth is Patterson *et al*. (2016), where an SEM figure of just *D. exilis* is shown. It shows that the abscission zone for *Digitaria* can be found at the base of the spikelet, similar to other closely related millet crops. This can be seen in our results, with both the shattered and complete spikelets. Abscission zones are known to form differently across the grasses, with differential cell size and lignification being two strategies for formation, regulated by genes including *sh1*, *sh4*, *qSH3* and *qShi1* (Yu and Kellogg, 2018; Yu *et al*., 2020; Mascher *et al*., 2024). Differential cell shapes appear to be important to abscission zone formation in *Digitaria*, though there is no obvious difference in formation between wild and cultivated species. In the assembly of the fonio genome (with surveys for domestication signatures), Abrouk *et al*. (2020) find that only 37 % of accessions of fonio across its whole cultivated range have a deletion in the *sh1* gene, and that plants with the deletion only show a 7 % chance of reduced shattering. Although this deletion has not been identified in the wild relative *D. longiflora*, this is a low expression of non-shattering phenotypes even if the gene is under selection.

In a survey of major plant domestication pathways (Fuller *et al*., 2023), the domestication centre of white fonio is placed in the Inner Niger Basin, Mali, with African rice, pearl millet and cowpea, but only fonio is described as having been domesticated through a ‘segetal pathway’ versus a ‘grain pathway’. Segetal is defined as a ‘former weedy species that grew in agricultural contexts that were added to the crop repertoire’. These segetal domesticates, which grew as weeds and were adopted into mainstream cultivation through convenience, were thought to be domesticated much later, and more quickly than others, likely around 2000–3000 years ago. Other ‘segetal pathway’ crops, including rye and oats, probably grew as weeds in fields of wheat and barley before selection. For fonio, a suggestion is that these harbour crops would have been African rice and pearl millet in the Mali region. Pearl millet was domesticated much earlier in Mali, and was very popular until African rice, fonio and sorghum were later incorporated into local food selection (Champion *et al*., 2021). This explains why fonio may have poorly developed domestication traits. A genetic resources and breeding programme study of fonio in Nigeria and Benin (Kanlindogbe *et al*., 2020) agrees that across West Africa there has been little conscious, targeted selection of fonio cultivars, describing cultivated varieties as ‘ecotypes’. In Burton *et al*. (2024), farmers interviewed in Guinea explained that landraces were only selected for suitability to local microclimates, which changed in rotations every few years, and not for specific uses, suggesting a low domestication pressure for morphological traits, and explaining why traits like shattering have not been significantly improved. Another factor may be the co-existence of *D. exilis* and *D. longiflora* often in the same fields, noted in Guinea, with overlapping occurrence ranges across West Africa (Abrouk *et al*., 2020; POWO, 2025). Though low (around 2 %; Barnaud *et al*., 2017) outcrossing in *D. exilis* plants has been observed, introgressions between crop and wild species are still likely, effectively slowing the rate of domestication pressure. This adds weight to the theory that cereal domestication can be a long, slow, ongoing landscape-level process, with no single epicentre for some species (Allaby *et al*., 2017, 2022).

### Conclusions

This study presents a comprehensive, widely sampled phylogeny of the grass genus *Digitaria*, including all four lineages domesticated as food crops (and their wild relatives) throughout history. Tree topologies are largely congruent with cladistics presented by previous studies, and three domesticated crops (white and black fonio and raishan) appear as distinct, monophyletic lineages, while the European Polish millet appears to be part of a more complicated taxonomic complex. Type specimens of the rare crops Indian-endemic raishan and West African black fonio are sequenced and presented here in phylogenetic analyses for the first time. The closest wild relatives to raishan are found to be wild Asian native species *D. cruciata*, *D. radicosa* and *D. setigera*. New close relatives for both fonio species are identified in *D. atrofusca* (for black fonio) and *D. fuscescens* and *D. argyrotricha* (for white fonio), all African native species. We also confirm through communication with specialists in India and Nigeria that *D. iburua* and *D. compacta* were still cultivated in 2025.

As the degradation of African grasslands, including large areas of millet cultivation, accelerates due to human activity and climate change, causing erratic weather patterns and increased fire activity, it will be vital to invest in crops that are resilient to fire and extreme climates (Osborne *et al*., 2018; Joseph *et al*., 2024; Knowles *et al*., 2025). *Digitaria* species, not only white and black fonio, have the evolutionary history and genetics likely to hold potential for improving food security for vulnerable areas in the future.

Future priorities for research should include collections of fresh and dried plant and seed material for both raishan and black fonio with ethnobotanic data, as seed-banking and conservation of traditional crops and knowledge alongside indigenous peoples are vital tools that will help to prevent the ongoing loss of these rare traditional crops.

## Supplementary Material

mcaf212_Supplementary_Data

## SUPPLEMENTARY DATA

Supplementary data are available at *Annals of Botany* online and consist of the following. Figure S1: morphological features of *Digitaria* grasses. From top left clockwise: inflorescence and racemes; spikelets; rachis and pedicels; and growth forms. Figure S2: photograph of a farmer in the Khasi Hills, India, with the millet raishan (*Digitaria compacta*). Reproduced from Singh and Arora (1972). Figure S3: morphological traits including spikelet arrangements (binate versus ternate) in (a) and spikelet hair type (complex, simple or mostly glabrous) in (b), across ML phylogeny of *Digitaria* species. Figure S4: ploidy estimations produced by nQuire, given as 2 (diploid), 3 (triploid) and 4 (tetraploid), across ML phylogeny. Figure S5: ancestral biogeographic origin of *Digitaria* species, produced by BioGeoBEARS analysis. Lineages are coloured by regions of native distribution from A to E. Clades corresponding to the four crop species are indicated. Figure S6: scanning electron microscope photos of *Digitaria* abscission zones in wild species. Captured at Kew Gardens and the Natural History Museum (UK). Red arrows indicate the abscission zone. From top left to bottom right: *D. abyssinica* (Schimper 82), *D. argyrotricha* (Scholz, H. 1096), *D. compressa* (A. Blair Rains 207), *D. debilis* (X.M. van der Burgt 1771), *D. diagonalis* (I. Darbyshire 471), *D. gayana* (F.F. Merklinger 15), *D. perrotettii* (F.F. Merklinger 104) and *D. setigera* (Clayton 5338). Figure S7: growth form morphologies of *Digitaria* species for cultivated crops (row 1), wild relatives (rows 2 and 3) and early-diverging fire-adapted species (row 4). Photographs are to the same scale, using the scale bar in the bottom left image. Table S1: details of specimens sampled for DNA extraction and phylogenetic analysis. Table S2: DNA sequence recovery statistics from HybPiper assembly analysis.
